# Supplementary material for: Palm fruit colours are linked to the broad-scale distribution and diversification of primate colour vision systems
Source: Proc Biol Sci. 2020 Feb 26;287(1921):20192731. doi: 10.1098/rspb.2019.2731 (PMC7062032; doi:10.1098/rspb.2019.2731)
Supplement: Supplemental Information methods, tables and figures [file rspb20192731supp1.pdf]

Supplementary Information

Palm fruit colours are linked to the broad-scale distribution and diversification of primate colour vision systems

Renske E. Onstein[1,2], Daphne N. Vink[2], Jorin Veen[2], Christopher D. Barratt[1,3], Suzette G.A. Flantua[2,4], Serge A. Wich[2,5], W. Daniel Kissling[2]

[1] German Centre for Integrative Biodiversity Research (iDiv) Halle-Jena-Leipzig, Deutscher Platz 5e, 04103 Leipzig, Germany.
[2] University of Amsterdam, Institute for Biodiversity and Ecosystem Dynamics (IBED), P.O. Box 94240, Amsterdam, The Netherlands.
[3] Max Planck Institute for Evolutionary Anthropology, Department of Primatology, Deutscher Platz 6, 04103 Leipzig, Germany.
[4] University of Bergen, Department of Biological Sciences, PO Box 7803, 5020, Bergen, Norway.
[5] Liverpool John Moores University, School of Natural Sciences and Psychology. Byrom street, L33AF, Liverpool, UK.

*Corresponding author: onsteinre@gmail.com

Proceedings of the Royal Society:B, doi:10.1098/rspb.2019.2731

Supplementary methods

Primate data

We collected, inferred or interpolated data on functional traits (colour vision, diet and activity level), species distributions and phylogenetic relationships for 411 primate species (100% of total species assessed for the International Union for Conservation of Nature's Red List 2017). For the colour vision data, we classified species as routine trichromatic, polymorphic or non-trichromatic (i.e. monochromatic or dichromatic) based on 15 studies from the primary scientific literature (see Appendix S1). Polymorphic primates have males with dichromatic vision and females with dichromatic or trichromatic vision. Six primate species were excluded because their vision data was missing, and therefore 405 primate species remained. For 90 primate species, vision data was available at the family level, for 295 species at the genus level, and for 17 species at the species level. Moreover, three primate species *(Cacajao ayresi, C. hosomi* and *C. melanocephalus)* were interpolated with vision data from a species in their genus.

For diet, we classified frugivores as those primates that include fruits as part of their diet (using the 1–3 ranking in the MammalDIET dataset, the ranking indicates the relative importance of fruits in the diet as compared to other food types) (Kissling et al., 2014, for 95% matching the recent update by Gainsbury et al., 2018). For activity status, we collected data on whether a species is day-active or not (i.e. night-active or crepuscular) from the EltonTraits 1.0 database (317 primate species, Wilman et al., 2014), supplemented with data from the Handbook of the Mammals of the World: Primates (61 species, Mittermeier et al., 2013). For 27 primate species the activity data were interpolated from the genus. These species belong to eight genera, and in all of these genera the activity level is conserved (see Appendix S1). This resulted in n = 158 routine trichromatic primates (n = 126 species of day-active, frugivores trichromatic primates, i.e. 31% of total primates) and n = 126 polymorphic primates (all polymorphic primates are day-active frugivores). All trait data and references are available from Appendix S1 and Dryad 10.5061/dryad.6hdr7sqwn.

Palm data

From a total of 2557 palm species (following the World Checklist of palms, Govaerts and Dransfield, 2005), ripe fruit colour data were assembled for 1749 species (c. 70% of total) from species descriptions in primary literature, monographs, the e-monocot database and from herbaria (Royal Botanic Gardens Kew Herbarium [K], Aarhus University Herbarium [AAU]) (Kissling et al., 2019). Fruit colours can be classified as 'non-conspicuous' when light reflectance spectra of fruits are similar to leaves, whereas fruits can be classified as 'conspicuous' when spectra differ between fruits and leaves (Regan et al., 2001). Since availability of spectral measurements is limited, we measured fruit reflectance spectra from 54 fresh palm fruits belonging to 18 species and compared them to qualitative colour descriptions from the literature (for details see below). This confirmed that colour descriptions of conspicuous fruits based on human vision match up with measured fruit reflectance data (see Table S3 and Fig. S16). This is also supported by fruit reflectance data from a wider variety of plant species (Sinnott-Armstrong et al., 2018). Following (Dominy et al., 2003), we then classified orange, red, yellow and pink fruits as conspicuous, and brown, black, green, blue, cream, grey, ivory, straw-coloured, white and purple fruits as non-conspicuous. Although purple fruits could be seen as conspicuous, both dichromats and trichromats can distinguish or detect the 'dark' colour against the background, and here they were thus included in the 'non-conspicuous' classification. If a fruit was described as a

combination of non-conspicuous and conspicuous colours (e.g. 'green/yellow', 'yellow-brown', 'brown orange') then the non-conspicuous colour was the dominant hue and the fruit colour was classified as non-conspicuous. Colours that were described with a suffix *-ish* or *-ey* were considered to have only a touch of that colour. Primates mostly feed on brown, green, orange, yellow, red and purple fruits (Fleming and Kress, 2013). Therefore, we excluded palm fruits that did not have these colours in their description from the analyses, and only included the species with brown, green, orange, yellow, red or purple fruit colours (n = 1444 palm species remained). All trait data, criteria and references are available from Appendix S2 and Dryad 10.5061/dryad.6hdr7sqwn.

Fruit colour experiment

Ideally, fruit colours would be quantified as fruit reflectance and modelled according to trichromatic primate visual phenotypes, instead of following an assignment derived from human vision. However, the paucity of spectral measurements limits our ability to assess broad-scale patterns in fruit colours based on such measurements. Nevertheless, to validate our results using qualitative colour descriptions to assign fruits to the 'conspicuous' and 'non-conspicuous' categories, we compared them to quantitative variables derived from measuring fruit reflectance spectra as red, green and blue (RGB) hues from 54 fresh palm fruits belonging to 18 species. These species are cultivated and were collected in Xishuangbanna Tropical Botanical Garden (Yunnan, China) in August 2017. These species come from different phylogenetic lineages and thus broadly represent the taxonomic diversity of palms, and also represent palms from all geographical realms (see Table S2, 'Natural region'). These species were also selected to represent the range of fruit colours observed in palms (i.e. red, orange, yellow, green, black). Fruit colours were quantified by photographing the fruits (using Nikon's macro lens AF-S VR Micro-Nikkor 105mm f/2.8G IF-ED) under standardized light settings and the angle of illumination and reflection was fixed at 45° to minimize glare. We quantified RGB hues from the photographs in Adobe Photoshop. Results showed that conspicuous fruits can be mostly quantified as those with at least as much red as green in the fruits, and at least as much red as 1.5 times the amount of blue (see Table S3 and Fig. S16). This suggests that the human perception of fruit colour as the basis of our study is both reliable and quantifiable.

Structural equation modelling

We started our modelling process with an *a priori* SEM that included all hypothesized pathways among all predictor variables (Fig. S1). Thus, we examined direct effects of climate (annual mean temperature, annual precipitation, temperature seasonality and precipitation seasonality) on forest heights and on the proportion of conspicuous palm fruits, respectively. We further examined direct effects of climate, forest height, area, and the proportion of conspicuous palm fruits on trichromat richness. In the next step, we evaluated the model's modification indices, model fits and residual correlations (Grace et al., 2012). We used the chi-square test, the root mean square error of approximation (RMSEA), and the comparative fit index (CFI) to measure the fit of the model. To ensure an adequate fit of SEMs we applied the following criteria: P-values of chi-square tests > 0.05, CFI > 0.90 and confidence intervals of RMSEA < 0.05 (Grace et al., 2012). The path with the least statistical significance was deleted from the *a priori* SEM and this model was then assessed to identify the next, least significant path. We repeated these steps until our final SEM only consisted of significant pathways (at $p < 0.05$), for which we extracted the standardized coefficients.

We repeated the global SEM procedure separately for the three biogeographical

realms (Africa, Americas, Asia) to evaluate whether these places differ in the relationship of colour vision with conspicuous fruits. The number of included botanical countries varied per continent (for day-active, frugivorous routine trichromats; Africa: 39 botanical countries, Americas: 26 botanical countries, Asia: 24 botanical countries, for day-active, frugivorous polymorphic primates: Africa: 1 botanical country, Americas: 29 botanical countries, Asia = 0 botanical countries). Analyses with $n = 1$ (e.g. African polymorphic primates only occur on Madagascar) or $n = 0$ (e.g. no polymorphic primates in Asia) botanical countries were not performed.

Sensitivity analyses

To evaluate whether an association between colour vision and fruit colour could result from confounding correlations, we performed five sensitivity analyses, and an additional analysis when also including figs (for details see last section in Supplementary methods). First, we repeated the SEMs but used the diversity of non-frugivorous trichromatic and/or polymorphic primates (instead of all or only day-active, frugivorous trichromatic primates) as the response variable. Under this scenario, we expected that the proportion of conspicuous palm fruits would not (significantly) explain species richness of non-frugivorous (mainly leaf-eating or 'folivorous') trichromats/polymorphs, suggesting that our results are not simply driven by confounding correlations between conspicuous fruits and other variables. Second, we evaluated the effect of the proportion of conspicuous fruits on dichromat species richness. Dichromatic species richness is expected to not be influenced by conspicuous fruits. Third, we evaluated whether differences in total trichromat/polymorphic species richness between biogeographical realms (trichromats/polymorphs, Americas $n = 14/113$, Africa $n = 62/14$, Asia $n = 82/0$) may have (erroneously) influenced our results. To this end, we divided trichromatic and/or polymorphic primate species richness in each botanical country by the total primate species richness in the respective continent, and repeated the global SEM. Fourth, we repeated the SEMs but followed the classification of frugivory from Galán-Acedo et al. (2019) (also available from Appendix S1), which only identifies frugivores as those species that predominantly feed on fruits. In these data, 39 routine trichromatic primate species and 36 polymorphic species which were classified as frugivorous (because fruits represent at least a small part of their diets) were classified as non-frugivorous (e.g. in genera *Alouatta, Trachypithecus* and *Chlorocebus, Cebus, Mico* and *Saguinus*). An additional six species (mainly in genus *Macaca*) were described as frugivorous whereas in our data they were classified as non-frugivorous. We expected that these differences in diet classification would not significantly affect our results. Fifth, we assessed whether our results were consistent with using conspicuous palm fruit species richness (rather than the proportion of conspicuous palm fruits) to explain primate species richness, and whether we fail to find a relationship between non-conspicuous palm fruit species richness and trichromatic and/or polymorphic primate species richness. The rationale for using conspicuous fruit proportions is that it allows for a direct comparison with non-conspicuous fruits, whereas food plant species richness could, potentially, be associated with primate species richness due to other, unmeasured, variables. Results of these sensitivity analyses are provided in Table S2.

Spatial autocorrelation

Spatial autocorrelation can affect significance tests and coefficient estimates in our SEMs (Kissling and Carl, 2008, Legendre and Legendre, 1998). We assessed the impact of spatial autocorrelation on our results by fitting spatial autoregressive (SAR) models, which allows the inclusion of residual spatial autocorrelation (Kissling and Carl, 2008). First, we fitted non-

spatial, ordinary least squares (OLS) regression models with the same set of predictor variables on trichromat richness as recovered from the SEMs. As standardized coefficients from the OLS models are equivalent to the path coefficients of the SEMs, they enable a direct comparison of the spatial autocorrelation between the spatial and non-spatial models (Kissling et al., 2008). The spatial weights matrix for the SAR models was defined using the minimum distance that linked each occupied grid cell to at least one other occupied cell (i.e. 326 km). To identify the extent of spatial autocorrelation in our data, we used correlograms and visualized changes in Moran's *I* values in the raw data response variables, the residuals of the non-spatial OLS and the SAR models. The standardized coefficients from the SAR models were very similar to those of the OLS models. We therefore focused on the path coefficients from the SEMs. The OLS model residuals were identified using the R package ncf and the spatial weight matrices of the SARs and the Moran's *I* values were calculated using the R package 'spdep' (Bivand and Wong, 2018).

Spatial autocorrelation did not strongly affect the results. For the global analysis, the standardized coefficient for the effect of conspicuous fruits on polymorphic primate richness obtained from the SAR model (std. coeff. = 0.21) was similar to the coefficient obtained from using the (non-spatial) SEM (Fig. 2a). Similarly, the standardized coefficient for the effect of conspicuous fruits on routine trichromat richness obtained from the SAR model (std. coeff. = 0.43) was similar to the coefficient obtained from using the (non-spatial) SEM (Fig. 2b). However, the positive effect of precipitation seasonality on trichromat richness became non-significant in the SAR model ($p = 0.54$) as compared to the SEM. Spatial autocorrelation did not significantly influence the SEMs performed using botanical countries for the different biogeographical realms (Africa, America, Asia), but did affect the Africa grid-based analysis (when including palms only). In Africa, the (positive) effect of conspicuous palm fruits on trichromat richness remained significant in the SAR model (std. coeff. = 0.091, $p < 0.001$), although the standardized coefficient was lower than the coefficient obtained from using the (non-spatial) SEM (Fig. 2c). Temperature and precipitation did not significantly affect trichromat richness in the SAR model ($p = 0.17$ and $p = 0.08$, respectively).

Ancestral colour vision and fruit colour reconstructions

We reconstructed the origin of trichromacy/polymoprhy and conspicuous fruits in primates and palms, respectively, by using stochastic character mapping (Huelsenbeck et al., 2003). This was done by sampling 500 stochastic character maps from the posterior probability distribution using a Markov chain Monte Carlo under the unequal transition rate model (i.e. the evolutionary transition rate of losing trichromacy, and losing conspicuous fruit colours, is different from the transition rate gaining trichromacy, or gaining conspicuous fruit colours, respectively). This resulted in a sample of unambiguous histories for trichromatic/polymorphic colour vision and conspicuous fruits over the primate and palm phylogenies, respectively. We summarized these by showing the posterior probability that the edges and nodes of the tree are inferred to have trichromatic colour version (as compared to non-trichromatic vision) or conspicuous fruits (as compared to non-conspicuous fruits) (see Fig. S11).

Analyses with figs

We used palms as a proxy for primate food plants, because comprehensive global trait, distribution and phylogenetic data for other keystone plant clades of primates are not available. However, we evaluated whether our results for Africa based on palms were consistent with another keystone food plant clade, namely figs (*Ficus*, Moraceae) (Shanahan

et al., 2001) for which good functional trait and distribution data are available for mainland Africa (Kissling et al., 2007).

Fruit colour data were assembled for all 86 African fig species (100% of figs on African mainland, thus excluding Madagascar) from the FigWeb of the Iziko Museums of Cape Town (http://www.figweb.org/Ficus/Species_index/afrotropical_species.htm). Species distribution maps of all 86 African figs were based on expert-based range maps provided by the Iziko Museums of Cape Town (for details see Kissling et al., 2007). For the phylogenetic data, we pruned the fossil-calibrated Moraceae MCC phylogenetic tree from Zhang et al. (2018) to include only African figs (n = 34 species, i.e. 52 African fig species with trait data were missing from the phylogeny).

We repeated the African SEM as well as the ancestral diversity reconstructions on the African fig phylogenetic tree. Results were consistent across palms and figs, however, the SEMs including figs showed weaker effects of the proportion of conspicuous fruits on trichromatic primate species richness as compared to palms only (Table S2). This effect became non-significant when correcting for spatial autocorrelation in model residuals (p = 0.1). Nevertheless, including figs provided a pattern similar to palms only regarding the standardized coefficient (obtained from SEMs) of the proportion of conspicuous food plant species on trichromat richness from arid to tropical climates, peaking in subtropical regions (compare Fig. S9 to Fig. 3). Furthermore, African figs with conspicuous fruits (as compared to those with non-conspicuous fruits) show rapid diversity increases from c. 10 Ma (Fig. S13), similar to the result obtained for palms (Fig. 4).

In conclusion, the results including figs indicate that fig fruits probably played a less important role for trichromat distribution and diversification in mainland Africa than palm fruits (also see Dominy et al., 2003).

Table S1: Summary statistics of trichromatic and polymorphic primates, palm fruit colours and climate variables for different regions and realms. Numbers indicate average values across the botanical countries in the region. Regions follow the classification from TDWG. Generally, Africa shows the highest trichromat species richness and the highest proportions of conspicuous palm fruits, followed by Asia and the Americas. Prec. = precipitation, Temp. = temperature, Seas. = seasonality, # = number of countries within region, mm = millimetre, m = meter.

Region (TDWG)	Realm	#Botanical Countries	Routine trichromat richness	Polymorphic primate richness	Palm richness	% Conspicuous	% Non-conspicuous	Prec. (mm)	Prec. Seas. (coefficient of variation in mm)	Temp. (°C * 10)	Temp. Seas. (annual range in °C * 10)	Forest height (m)
West-Central Tropical Africa	Africa	10	13.4	0	19	0.59	0.41	1618	63	230	881	18.5
East Tropical Africa	Africa	3	12.3	0	7.3	0.51	0.49	927	73	231	1009	14.4
Asia-China	Asia	5	10	0	79.4	0.41	0.59	1788	85	244	2320	23.5
West Tropical Africa	Africa	14	9.6	0	12.2	0.59	0.41	1258	102	268	1991	9.4
Malesia	Asia	6	7.2	0	93.5	0.38	0.62	2430	41	250	477	20.4
Northeast Tropical Africa	Africa	4	6.8	0	5.5	0.26	0.74	448	116	257	2402	9.1
China	Asia	4	4.8	0	25.8	0.55	0.45	1054	80	129	6132	27.9
Brazil	Americas	5	4.6	28.8	70	0.4	0.6	1555	59	231	1568	18.2
Western South America	Americas	4	4	26.8	126.5	0.41	0.59	1856	56	213	993	18.1
South Tropical Africa	Africa	5	3.4	0	7.2	0.37	0.63	959	101	219	2424	11.8
Indian Subcontinent	Asia	8	3.3	0	22.9	0.42	0.58	1463	89	193	4700	28.2
Southern Africa	Africa	3	2	0	3.7	0.47	0.53	610	63	177	3899	14.4
Southern South America	Americas	2	2	3.5	16	0.35	0.65	973	42	205	4048	8
Northern South America	Americas	4	1.8	9.5	61.8	0.48	0.52	2230	52	258	573	20.2
Mexico	Americas	3	1.3	1	44.3	0.13	0.87	1468	81	235	2042	21.2
Central America	Americas	7	1.3	23	63.6	0.15	0.85	2206	65	242	1023	21.2
Caribbean	Americas	1	1	1	21	0.38	0.62	2075	44	257	634	13.2
Eastern Asia	Asia	1	1	0	8	0.38	0.63	2523	70	191	3564	27.8

Table S2: Summary overview of sensitivity analyses. The base structural equations model (SEM) (Figure S1) was adjusted in terms of the response variable (i.e. different measures of primate species richness) and/or the explanatory variable (i.e. different measures of fruits), to assess how the standardized coefficient of fruits on primate richness changed as compared to the empirical results (Figures S2 and S7). Sensitivity SEMs were performed for the global dataset (with botanical countries) as well as for the mainland African grid dataset (110 × 110 km), and in most cases the analyses were performed for all primates vs. only day-active frugivorous primates. Results were also explored for trichromatic, polymorphic or trichromatic + polymorphic primates combined. 'Type' refers to the type of sensitivity analysis (A-G). (A) effect of proportion of conspicuous palm fruits on non-frugivorous primates as the response variable (no effect expected); (B) effect of proportion of conspicuous palm fruits on dichromatic primate species richness (no effect expected); (C) effect of conspicuous palm fruits on primate species richness, corrected for the continental differences in primate species richness (effect expected to be similar to empirical results, see Figure S2); (D) effect of conspicuous fruits on day-active frugivorous primate species richness in which the classification of frugivorous primates followed Galán-Acedo et al. (2019); (E1) effect of species richness (instead of proportion) of palms with conspicuous fruits on primate species richness (effect expected to be similar to empirical results, see Figure S2); (E2) effect of species richness of palms with non-conspicuous fruits on primate species richness (no or a negative effect expected); (F) effect of conspicuous fruits on primate species richness when also including figs in the model (effect expected to be similar to empirical results, see Figure S7). The last column indicates if the result supported our expectation. In case it did not, it applies to polymorphic primates for which we indeed did not find strong support for an association with conspicuous fruits (see main text), or when including figs, suggesting that these are not as closely associated with primate colour vision as expected (see main text). Last, although the effect of conspicuous fruits species richness on routine trichromatic primate species richness at the global scale was positive but not statistically supported, the effect was positive when focusing on Africa only, supporting our primary result (see main text). The complete results from the sensitivity analysis of SEMs (i.e. also including effects of climate and forest structure) are available on request from the first author. Tri = trichromatic primate species richness, Poly = polymorphic primate species richness, Tri+Poly = trichromatic and polymorphic species richness combined, PSR = primate species richness, All = all primates were included in the SEM, frugi = only day-active, frugivorous primates were included in the SEM, non-frugi = only non-frugivorous primates were included in the SEM, Consp. = proportion of conspicuous (palms, unless figs is indicated) fruits, Std. coeff. = standardized coefficient of the explanatory on the response variable as obtained with the SEM, None = no significant effect of the explanatory variable on the response variable was detected in the SEM, n.a. = not applicable.

Type	Continent	Response	All / Frugi / Non-frugi	Explanatory	Std. coeff. explanatory	Support expectation?
A	Global	Tri PSR	Non-frugi	Consp.	None	Yes
A	Global	Poly PSR	Non-frugi	Consp.	n.a. (all polymorphic primates are frugivores)	n.a.

A	Global	Tri+Poly PSR	Non-frugi	Consp.	None	Yes
A	Africa.grid	Tri PSR	Non-frugi	Consp.	None	Yes
B	Global	Dichromatic PSR	All	Consp.	None	Yes
B	Global	Dichromatic PSR	Frugi	Consp.	n.a. (too few data points - only Madagascar)	n.a.
C	Global	Tri / realm richness	All	Consp.	0.179	Yes
C	Global	Tri / realm richness	Frugi	Consp.	0.169	Yes
C	Global	Poly / realm richness	All	Consp.	None	No
C	Global	Poly / realm richness	Frugi	Consp.	None	No
C	Global	Tri+Poly / realm richness	All	Consp.	0.121	Yes
C	Global	Tri+Poly / realm richness	Frugi	Consp.	n.a. (all polymorphic primates are frugivores)	n.a.
D	Global	Tri	Frugi as classified by Galán-Acedo	Consp.	0.523	Yes
D	Global	Poly	Frugi as classified by Galán-Acedo	Consp.	0.272	Yes
D	Global	Tri + Poly	Frugi as classified by Galán-Acedo	Consp.	0.299	Yes
D	Africa.grid	Tri	Frugi as classified by Galán-Acedo	Consp.	0.332	Yes
E1	Global	Tri	All	Conspicuous fruit species richness	None	No
E1	Global	Tri	Frugi	Conspicuous fruit species richness	None	No
E1	Global	Poly	All	Conspicuous fruit species richness	0.393	Yes
E1	Global	Poly	Frugi	Conspicuous fruit species richness	n.a. (all polymorphic	Yes

					primates are frugivores)	
E1	Global	Tri + Poly	All	Conspicuous fruit species richness	0.319	Yes
E1	Global	Tri + Poly	Frugi	Conspicuous fruit species richness	0.332	Yes
E1	Africa.grid	Tri	All	Conspicuous fruit species richness	0.195	Yes
E1	Afria.grid	Tri	Frugi	Conspicuous fruit species richness	0.275	Yes
E2	Global	Tri	All	Non-conspicuous fruit species richness	-0.542	Yes
E2	Global	Tri	Frugi	Non-conspicuous fruit species richness	-0.309	Yes
E2	Global	Poly	All	Non-conspicuous fruit species richness	None	Yes
E2	Global	Poly	Frugi	Non-conspicuous fruit species richness	n.a. (all polymorphic primates are frugivores)	n.a.
E2	Global	Tri + Poly	All	Non-conspicuous fruit species richness	None	Yes
E2	Global	Tri + Poly	Frugi	Non-conspicuous fruit species richness	None	Yes
E2	Africa.grid	Tri	All	Non-conspicuous fruit species richness	None	Yes
E2	Afria.grid	Tri	Frugi	Non-conspicuous fruit species richness	None	Yes
F	Africa.grid	Tri	All	Palms + figs	None	No
F	Africa.grid	Tri	All	Figs only	None	No
F	Africa.grid	Tri	Frugi	Palms + figs	0.15	Yes
F	Africa.grid	Tri	Frugi	Figs only	0.062	Yes

Table S3: Fruit colour experiment. Measurements of red (R), green (G) and blue (B) in the reflectance spectra of palm fruits. The percentages R, G and B were calculated for 18 species (3 fruits per species), and matched against descriptions of fruit colours from the literature which were used to assign species to the non-conspicuous/conspicuous categories in this study. If fruits had more R than G in their reflectance, and more red than 1.5 times the amount of B, they were quantified as 'conspicuous'. If not, they were mostly 'non-conspicuous'. In the few cases in which the literature did not match the quantified colour (last column 'no'), the fruits were mostly immature.

Species	Biogeographic realm	%R	%G	%B	Non-conspicuous from literature	Conspicuous from literature	Does the colour have R > G and R > 1.5*B? Yes → Conspicuous No → Non-conspicuous	Does the colour measurement match the literature?
Acoelorrhaphe wrightii	Americas	39.5	33.9	26.6	1	0	no	yes
Acoelorrhaphe wrightii	Americas	35.4	34.7	29.9	1	0	no	yes
Acoelorrhaphe wrightii	Americas	35.3	35.3	29.4	1	0	no	yes
Areca concinna	Asia	65.4	16.8	17.8	0	1	yes	yes
Areca concinna	Asia	59.7	21.7	18.6	0	1	yes	yes
Areca concinna	Asia	47.3	28.7	24	0	1	yes	yes
Areca vestiaria	Asia	56.6	29.4	13.9	0	1	yes	yes
Areca vestiaria	Asia	54.8	28.3	17	0	1	yes	yes
Areca vestiaria	Asia	55.6	31.2	13.2	0	1	yes	yes
Arenga engleri	Asia	65.2	17.9	16.9	0	1	yes	yes
Arenga engleri	Asia	72.6	16.8	10.6	0	1	yes	yes
Arenga engleri	Asia	50.1	24.7	25.2	0	1	yes	yes
Arenga westerhoutii	Asia	36.6	39.6	23.8	1	0	no	yes
Arenga westerhoutii	Asia	36.5	39.5	23.9	1	0	no	yes
Arenga westerhoutii	Asia	36.3	37.9	25.9	1	0	no	yes
Carpentaria acuminata	Australasia	65.8	17.1	17.1	0	1	yes	yes
Carpentaria acuminata	Australasia	56.2	23	20.8	0	1	yes	yes
Carpentaria acuminata	Australasia	52.7	25.2	22.1	0	1	yes	yes
Cryosophila warscewiczii	Americas	42.9	44.8	12.3	1	0	no	yes
Cryosophila warscewiczii	Americas	42.2	44.23	13.5	1	0	no	yes
Cryosophila warscewiczii	Americas	38.6	40.9	20.5	1	0	no	yes
Dypsis lastelliana	Africa	37.3	34.3	28.4	n.a.	n.a.	no	n.a.
Dypsis lastelliana	Africa	34.6	35	30.5	n.a.	n.a.	no	n.a.
Dypsis lastelliana	Africa	37.7	34.9	27.4	n.a.	n.a.	no	n.a.
Dypsis lutescens	Africa	47.9	52.1	0	0	1	no	no
Dypsis lutescens	Africa	50.5	49.5	0	0	1	yes	yes
Dypsis lutescens	Africa	45.6	45.6	8.7	0	1	yes	yes

Hydriastele microspadix	Australasia	53.7	24.4	21.9	0	1	yes	yes
Hydriastele microspadix	Australasia	48	27	25	0	1	yes	yes
Hydriastele microspadix	Australasia	51	24.7	24.2	0	1	yes	yes
Latania verschaffeltii	Africa	37.6	41.2	21.2	1	0	no	yes
Latania verschaffeltii	Africa	39.5	39.7	20.8	1	0	no	yes
Latania verschaffeltii	Africa	36.4	39.4	24.2	1	0	no	yes
Licuala lauterbachii	Australasia	68.7	16.2	15.1	0	1	yes	yes
Licuala lauterbachii	Australasia	56.7	23.5	19.9	0	1	yes	yes
Licuala lauterbachii	Australasia	54.3	24.4	21.3	0	1	yes	yes
Livistona muelleri	Australasia	35.6	35.6	28.7	1	0	no	yes
Livistona muelleri	Australasia	34.4	35.5	30.1	1	0	no	yes
Livistona muelleri	Australasia	35.8	35.3	28.9	1	0	no	yes
Phoenix theophrasti	Europe	58	42	0	1	0	yes	no
Phoenix theophrasti	Europe	56.3	43.7	0	1	0	yes	no
Phoenix theophrasti	Europe	46.1	38.6	15.2	1	0	yes	no
Ptychosperma microcarpum	Australasia	68.4	12.8	18.8	0	1	yes	yes
Ptychosperma microcarpum	Australasia	70.4	12.3	17.3	0	1	yes	yes
Ptychosperma microcarpum	Australasia	69.5	14	16.51	0	1	yes	yes
Sabal blackburniana	Americas	33.9	35.7	30.4	1	0	no	yes
Sabal blackburniana	Americas	34.6	35.8	29.6	1	0	no	yes
Sabal blackburniana	Americas	39.9	42.1	18	1	0	no	yes
Sabal palmetto	Americas	46.8	53.2	0	1	0	no	yes
Sabal palmetto	Americas	44.8	50.7	4.5	1	0	no	yes
Sabal palmetto	Americas	47.5	52.5	0	1	0	no	yes
Syagrus romanzoffiana	Americas	41	37.2	21.8	0	1	yes	yes
Syagrus romanzoffiana	Americas	35.4	46.2	18.4	0	1	no	no
Syagrus romanzoffiana	Americas	36.4	43.5	20.1	0	1	no	no

Table S4: Summary overview of structural equation models (SEMs) in this study. SEMs were performed for different regions (global/America/Africa/Asia/Africa[grid]), different response variables (trichromatic, polymorphic or trichromatic/polymorphic primates combined) and different restrictions to the data (e.g., only focusing on frugivores or all primates). In addition, we show the standardized coefficient (from SEMs) of proportion of conspicuous fruits on primate richness, the coefficient in the spatial autoregressive (SAR) model if spatial autocorrelation affected the results, whether the effect of proportion of conspicuous fruits on primate richness was higher than any of the other direct effects (i.e., forest structure, area, temperature, precipitation, temperature seasonality and precipitation seasonality, see Fig. S1), which supplementary figure shows the result, and whether results were robust to simulations (i.e., 1000 randomisations of primates across spatial units and repeating the SEM - if the observed effect lied outside the 95% confidence interval of simulated effects, it was considered robust with respect to simulation). Tri = trichromatic primate species richness, Poly = polymorphic primate species richness, Tri+Poly = trichromatic and polymorphic species richness combined, All = all primates were included in the SEM, frugi = only day-active, frugivorous primates were included in the SEM, n.a. = not applicable.

Region	Response	All / Frugi	Std. coeff. conspicuous fruits	Spatial autocorrelation?	Largest effect of conspicuous fruits?	Fig.?	Robust to simulation?
Global	Tri+Poly	All	0.222	0.14, $p < 0.05$	No	S2	No
Global	Tri+Poly	Frugi	0.208	0.15, $p < 0.01$	No	S2	No
Global	Tri	All	0.547	0.45, $p < 0.001$	Yes	S2	Yes
Global	Tri	Frugi	0.554	0.43, $p < 0.001$	Yes	S2	Yes
Global	Poly	All = Frugi	0.171	0.21, $p < 0.01$	No	S2	No
Africa	Tri+Poly	All	None			S4	
Africa	Tri+Poly	Frugi	0.178		No	S4	
Africa	Tri	All	None			S4	
Africa	Tri	Frugi	0.289		No	S4	
Africa	Poly	All = Frugi	n.a. (only Madagascar)			n.a.	
America	Tri+Poly	All	None			S5	
America	Tri+Poly	Frugi	None			S5	
America	Tri	All	-0.534		No	S5	
America	Tri	Frugi	-0.577		No	S5	
America	Poly	All = Frugi	None			S5	
Asia	Tri	All	None			S6	
Asia	Tri	Frugi	None			S6	
Asia	Poly	All = Frugi	n.a. (no polymorphic primates)			n.a.	
Africa.grid	Tri = Tri + Poly	All	0.193	0.066, $p < 0.01$	No	S7	Almost
Africa.grid	Tri = Tri + Poly	Frugi	0.315	0.091, $p < 0.001$	Yes	S7	Yes

Figure S1: Structural equation model: the base model. The structural equation model (SEM) illustrates the direct and indirect effects of forest height, climate, area, and the proportion of conspicuous palm fruits on trichromat richness. Arrows indicate the direction of the effect. The red arrows highlight the relation between palm fruit colour and trichromat richness. Prec. = annual precipitation, Temp. = annual mean temperature, T Seas. = temperature seasonality, P Seas. = precipitation seasonality, Conspicuous fruits = proportion of conspicuous palm fruits, Trichromats = day-active, frugivorous, trichromatic primate species richness.

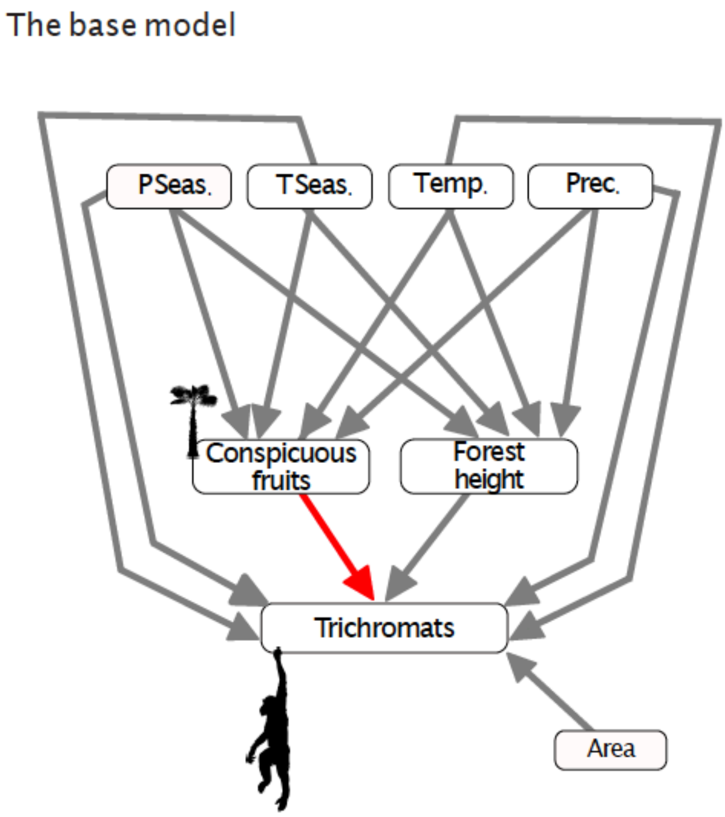

Figure S2: Determinants of trichromatic and polymorphic primate species richness. Structural equation models (SEMs) illustrate how the proportion of conspicuous palm fruits (red arrows) determines (a) trichromatic and polymorphic primate species richness, (b) day-active, frugivorous trichromatic and polymorphic primate species richness, (c) routine trichromatic primate species richness, (d) day-active, frugivorous routine trichromatic species richness, (e) polymorphic primate species richness and (f) day-active, frugivorous polymorphic primate species richness. SEMs were performed at the global scale (botanical country resolution). Effect sizes represent standardized coefficients and arrows indicate the direction of the effect, with arrow thickness being proportional to effect strength. Only statistically significant effects (at $p < 0.05$) are illustrated. Prec. = annual precipitation, Temp. = annual mean temperature, T Seas. = temperature seasonality, P Seas. = precipitation seasonality, Conspicuous fruits = proportion of conspicuous palm fruits, Tri+Poly = Trichromats + polymorphic primate species richness (combined), Trichromats = routine trichromatic primate species richness, Polymorphs = polymorphic primate species richness.

a. Fitted global model for all tri+polymorphs

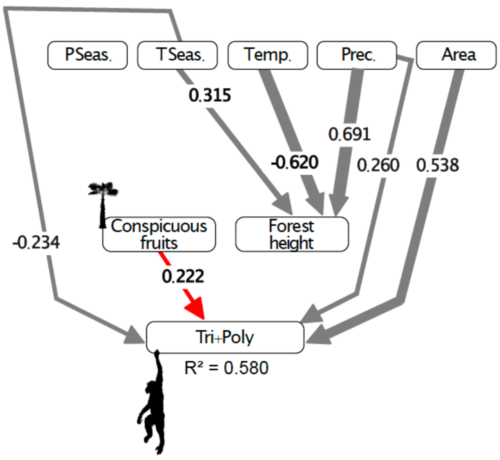

b. Fitted global model for frugivorous tri+polymorphs

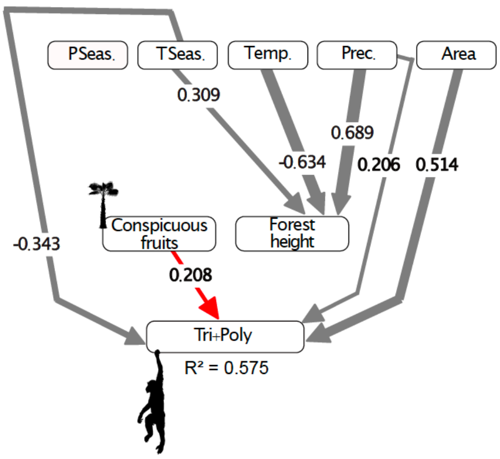

c. Fitted global model for all trichromats

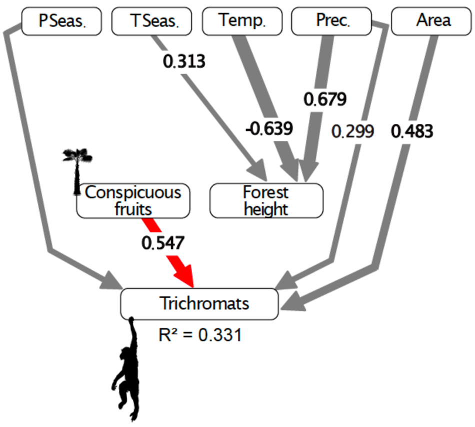

d. Fitted global model for frugivorous trichromats

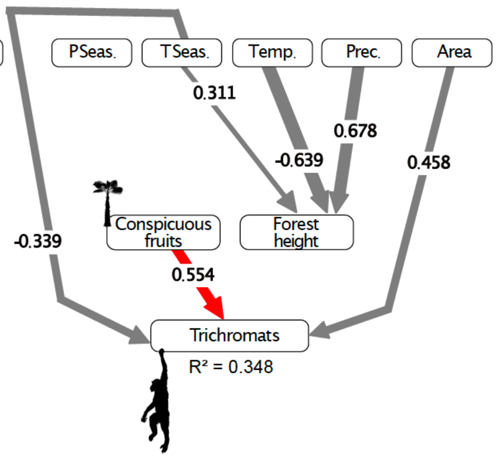

e. Fitted global model for all polymorphs

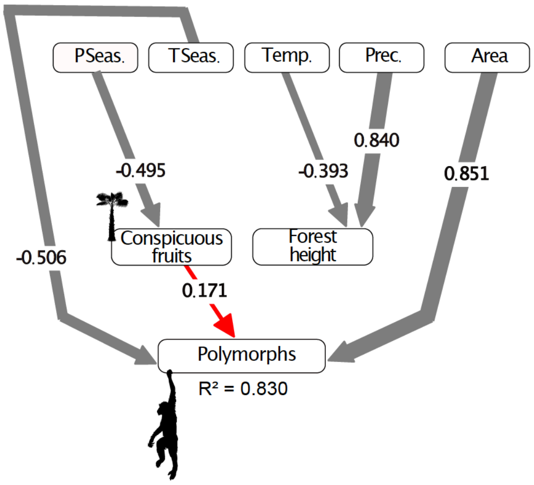

f. Fitted global model for frugivorous polymorphs

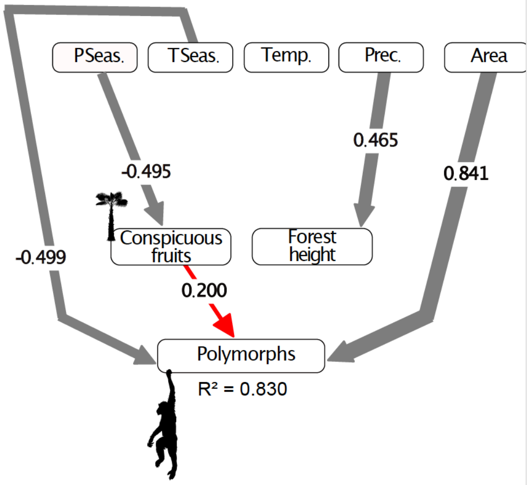

Figure S3: Simulations of trichromatic and polymorphic primates globally. We compared the observed standardized coefficient of proportion of conspicuous fruits on primate species richness as obtained from structural equation models (SEMs) to 1000 simulated effects. Simulations were performed by randomly assigning primate species (and their colour vision) to botanical countries, keeping the total species richness for each botanical country the same. The observed effect of conspicuous fruits on routine trichromat richness (c, d) is much higher than the effect expected under a random distribution, whereas the effect on polymorphic primate species richness (e, f) or routine trichromats and polymorphic primates combined (a, b) does not deviate from a random expectation.

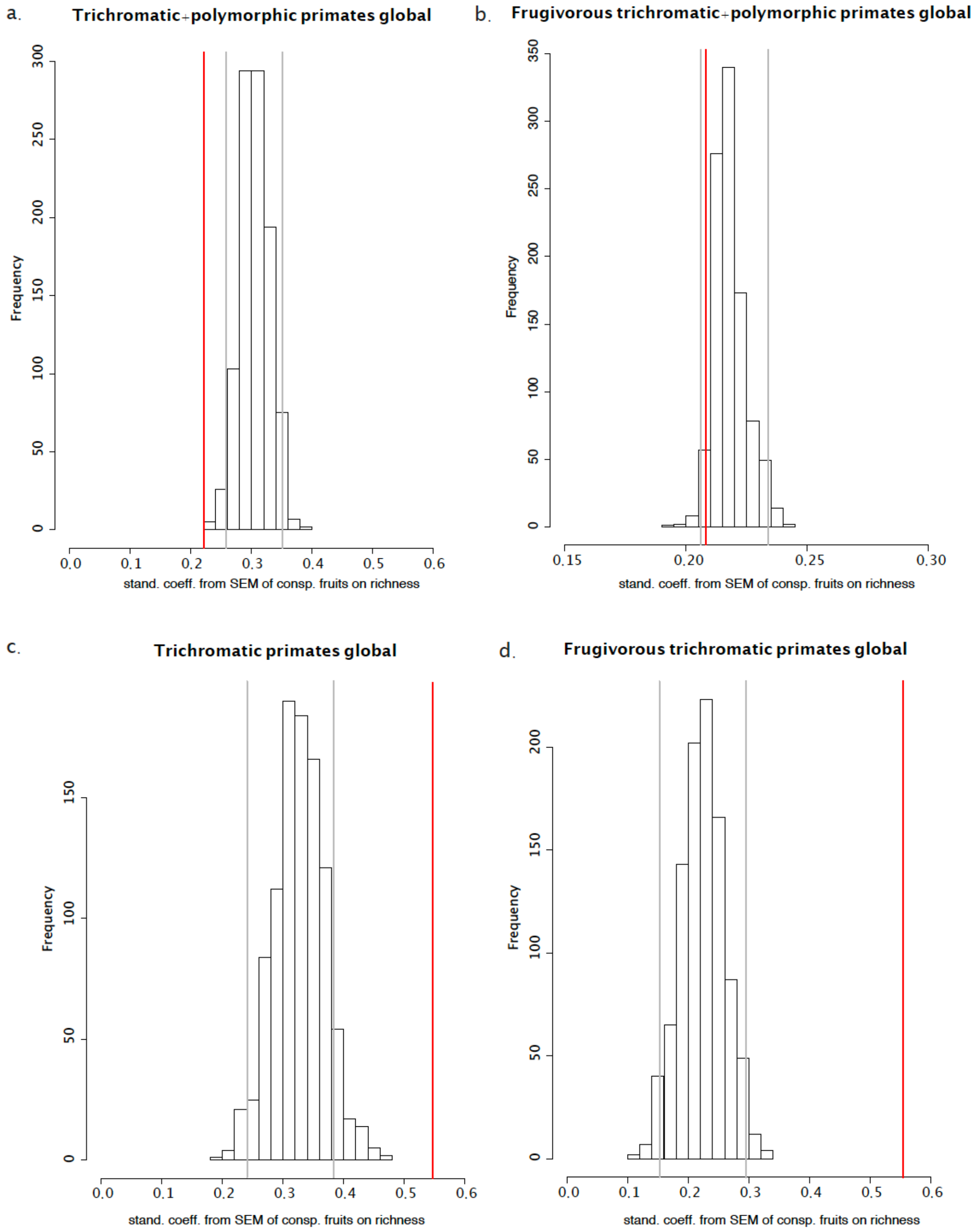

e.

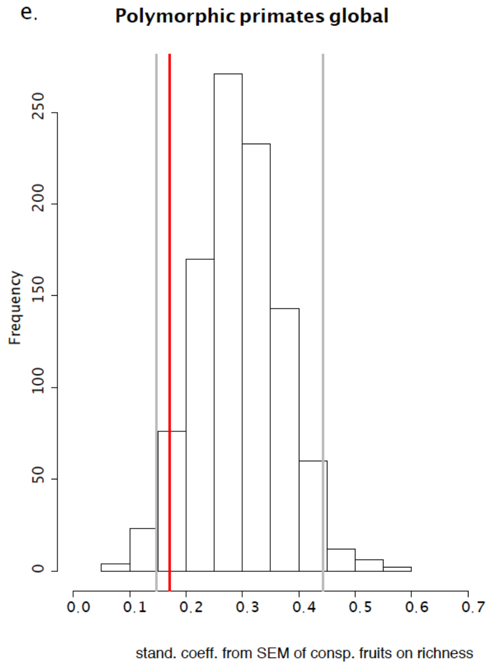
Polymorphic primates global
Frequency
0
50
100
150
200
250
0.0
0.1
0.2
0.3
0.4
0.5
0.6
0.7
stand. coeff. from SEM of consp. fruits on richness

f.

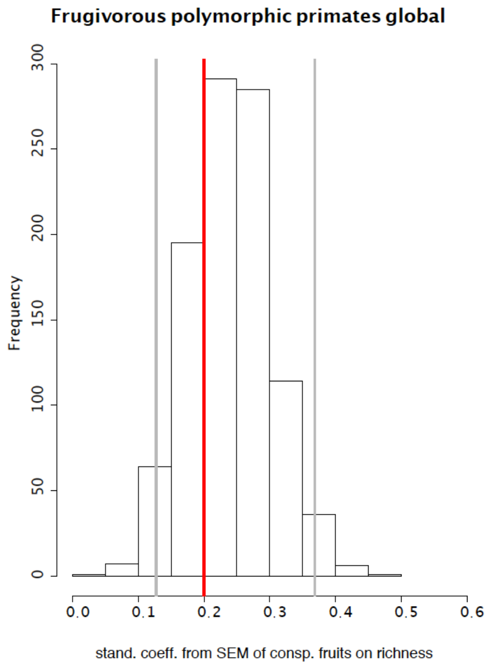
Frugivorous polymorphic primates global
Frequency
0
50
100
150
200
250
300
0.0
0.1
0.2
0.3
0.4
0.5
0.6
stand. coeff. from SEM of consp. fruits on richness

Figure S4: Structural equation models for Africa (botanical countries, including Madagascar). (a) all polymorphic and trichromatic primates, (b) day-active, frugivorous polymorphic and trichromatic primates, (c) all trichromatic primates, (d) day-active, frugivorous trichromatic primates. Please note that this is using botanical countries as spatial units whereas Fig. 2c (main text) illustrates the model using the fine resolution grid cells. SEMs for polymorphic primates only were not possible due to low sample size (*n* = 1, i.e. Madagascar). SEMs illustrate how the proportion of conspicuous palm fruits (red arrows) determines primate species richness once direct and indirect effects of forest height, climate, and area are accounted for. Effect sizes represent standardized coefficients and arrows indicate the direction of the effect, with arrow thickness being proportional to effect strength. Only statistically significant effects (at $p < 0.05$) are illustrated. Prec. = annual precipitation, Temp. = annual mean temperature, T Seas. = temperature seasonality, P Seas. = precipitation seasonality, Conspicuous fruits = proportion of conspicuous palm fruits, Poly+Trichromats = polymorphic and trichgromatic primate species richness, Trichromats = trichromatic primate species richness.

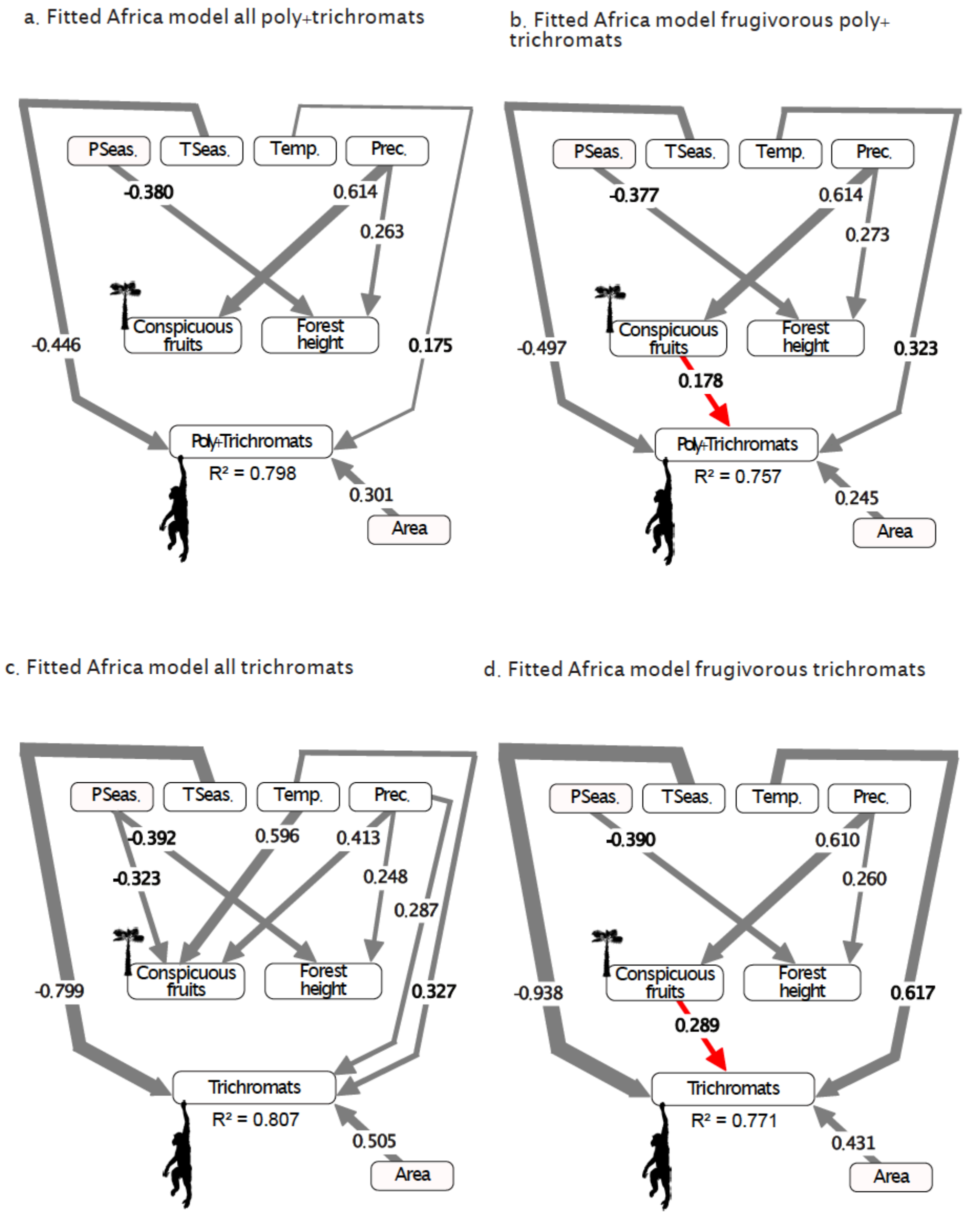

Figure S5: Structural equation models for the Americas. (a) all polymorphic and trichromatic primates, (b) day-active, frugivorous polymorphic and trichromatic primates, (c) all trichromatic primates, (d) day-active, frugivorous trichromatic primates, (e) all polymorphic primates, (f) day-active, frugivorous polymorphic primates. SEMs illustrate how the proportion of conspicuous palm fruits (red arrows) determines primate species richness once direct and indirect effects of forest height, climate, and area are accounted for. Effect sizes represent standardized coefficients and arrows indicate the direction of the effect, with arrow thickness being proportional to effect strength. Only statistically significant effects (at $p < 0.05$) are illustrated. Prec. = annual precipitation, Temp. = annual mean temperature, T Seas. = temperature seasonality, P Seas. = precipitation seasonality, Conspicuous fruits = proportion of conspicuous palm fruits, Poly+Trichromats = polymorphic and trichromatic primate species richness, Trichromats = trichromatic primate species richness, Polymorphs = polymorphic primate species richness.

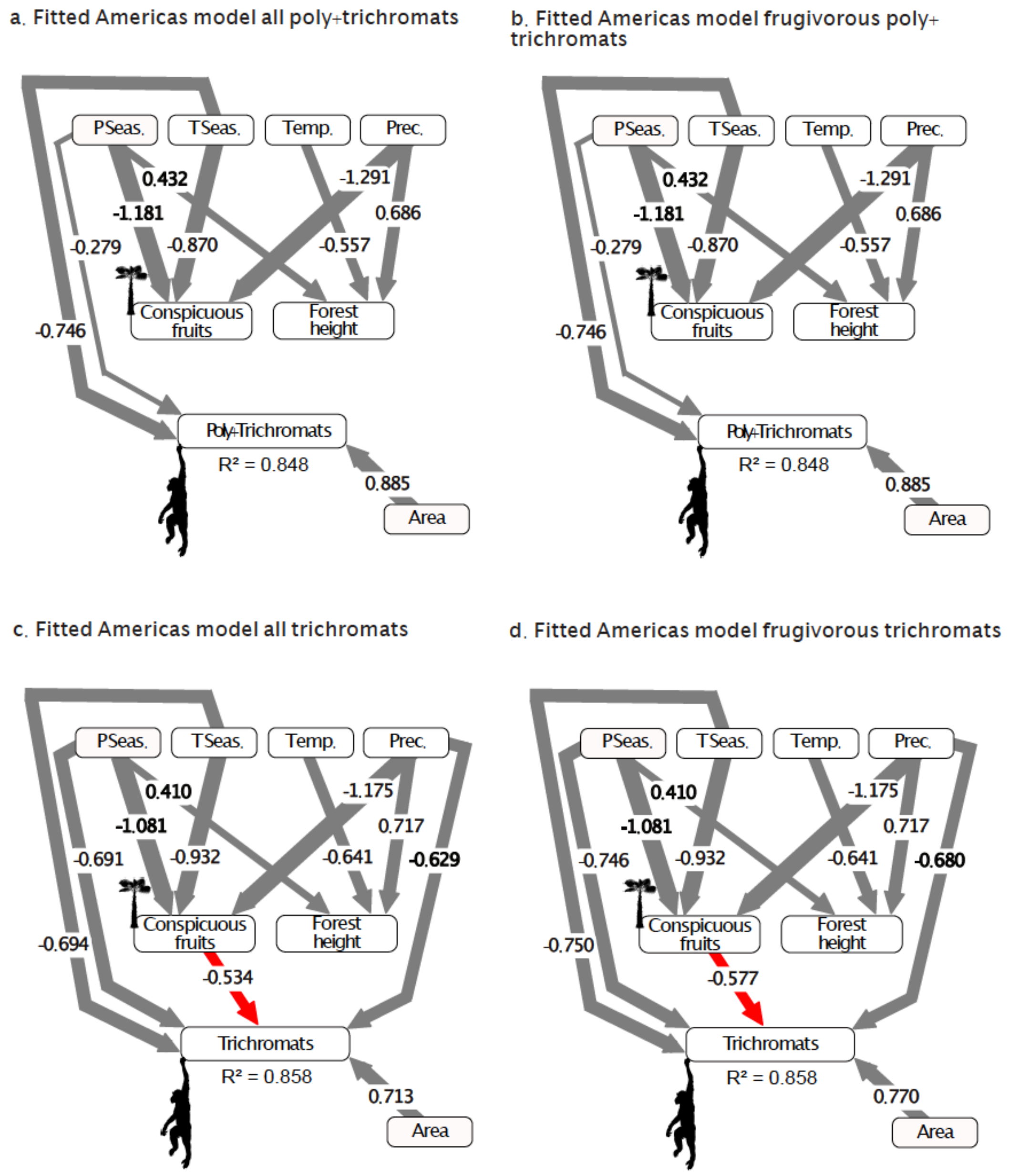

e. Fitted Americas model all polymorphs

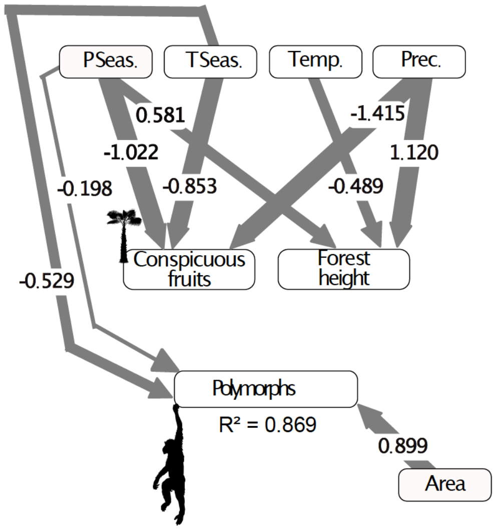

f. Fitted Americas model frugivorous polymorphs

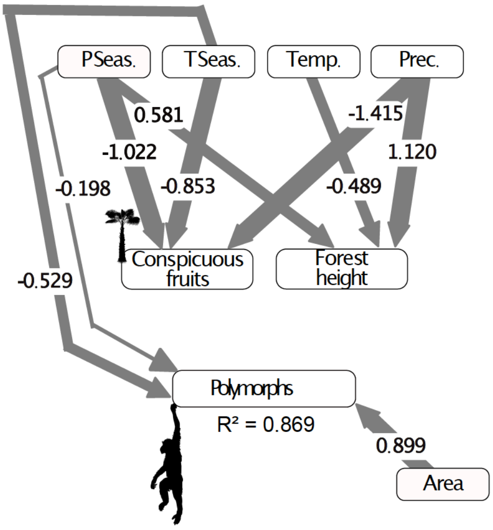

Figure S6: Structural equation models for Asia. (a) all trichromatic primates, (b) day-active, frugivorous trichromatic primates. Polymorphic primates are absent in Asia. SEMs illustrate how the proportion of conspicuous palm fruits (red arrows) determines primate species richness once direct and indirect effects of forest height, climate, and area are accounted for. Effect sizes represent standardized coefficients and arrows indicate the direction of the effect, with arrow thickness being proportional to effect strength. Only statistically significant effects (at $p < 0.05$) are illustrated. Prec. = annual precipitation, Temp. = annual mean temperature, T Seas. = temperature seasonality, P Seas. = precipitation seasonality, Conspicuous fruits = proportion of conspicuous palm fruits, Trichromats = trichromatic primate species richness.

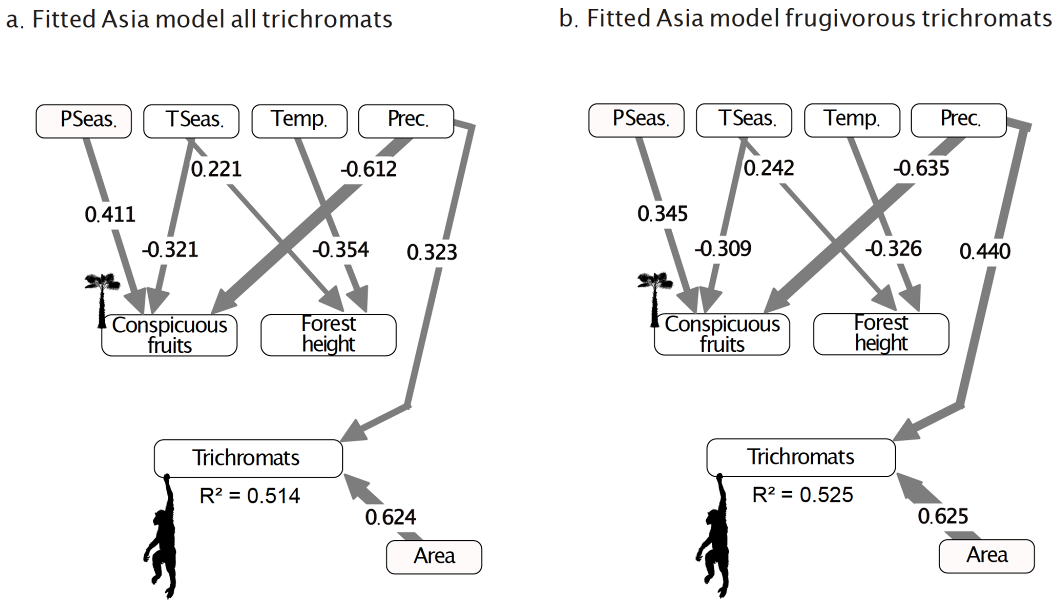

Figure S7: Determinants of trichromatic primate species richness in mainland Africa using grid cells. Structural equation models (SEMs) illustrate how the proportion of conspicuous palm fruits (red arrows) determines (a) trichromatic and (b) day-active, frugivorous trichromatic primate species richness across mainland Africa (where no polymorphic primates occur) (resolution of 110 × 110 km grid cells) once direct and indirect effects of forest height, climate, and area are accounted for. Effect sizes represent standardized coefficients and arrows indicate the direction of the effect, with arrow thickness being proportional to effect strength. Only statistically significant effects (at $p < 0.05$) are illustrated. Prec. = annual precipitation, Temp. = annual mean temperature, T Seas. = temperature seasonality, P Seas. = precipitation seasonality, Conspicuous fruits = proportion of conspicuous palm fruits, Trichromats = routine trichromatic primate species richness.

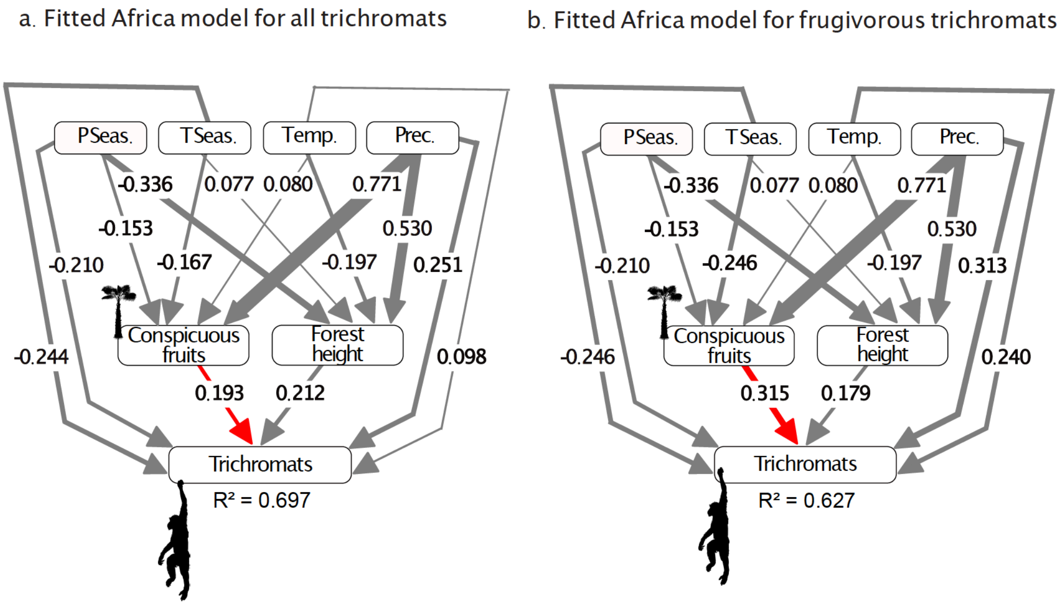

Figure S8: Simulations of trichromatic primates in mainland Africa. We compared the observed standardized coefficient of proportion of conspicuous fruits on trichromatic primate richness as obtained from structural equation models (SEMs) to 1000 simulated effects. Simulations were performed by randomly assigning primate species (and their colour vision) to African grid cells (*n* = 794 grid cells), keeping the total species richness for each grid cell the same. The observed effect of conspicuous fruits on routine trichromat richness is much higher than the effect expected under a random distribution.

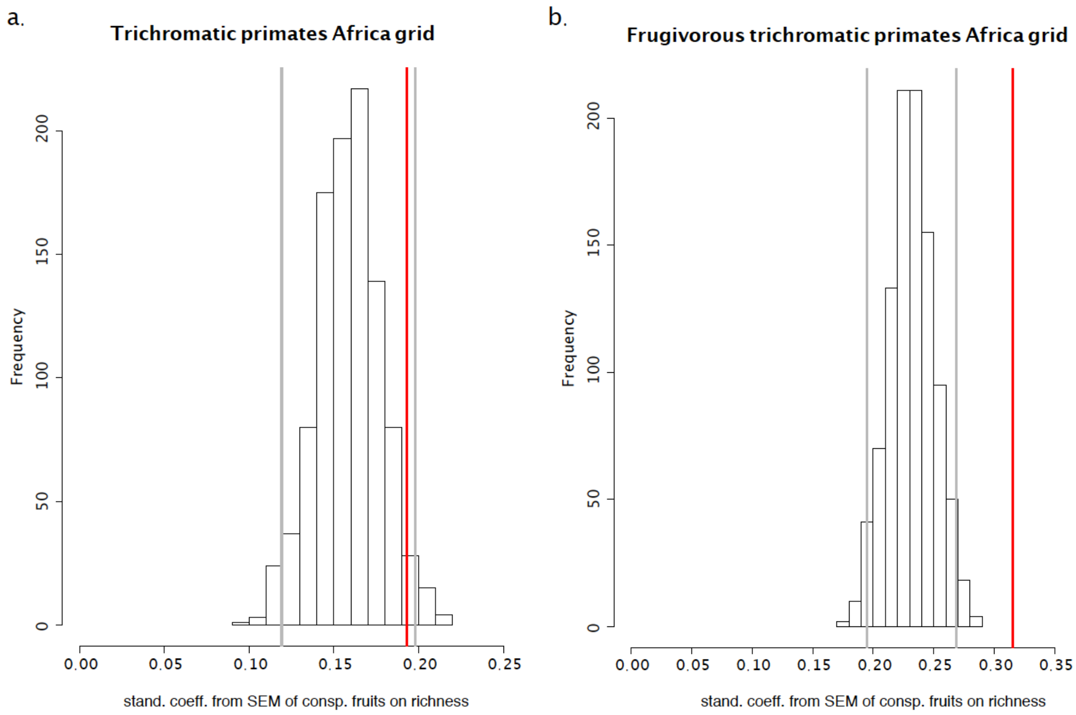

Figure S9: The importance of the proportion of conspicuous palm and fig fruits on trichromatic primate species richness along a gradient from arid to humid tropical climates in Africa. Standardized coefficients (±SD) representing the effect of the proportion of conspicuous fruits of palms and figs combined (a) and figs only (b) on day-active, frugivorous, trichromatic primate species richness, and of palms and figs combined (c) and palms only (d) on all primates. Standardized coefficients were obtained from structural equation models (SEMs) (*n* = 35 for palms and figs combined, *n* = 15 for figs only, *n* = 10 for palms only). These SEMs were fitted by progressively delimiting the number of included grid cells based on the minimum number of food plant species richness present (see Fig. S10 for spatial delimitation). Effect strength peaks in the subtropics at an intermediate food plant species richness. For figs only on all primates no significant effect was detected. See Figure 3 for palms only and the effect on day-active, frugivorous primates.

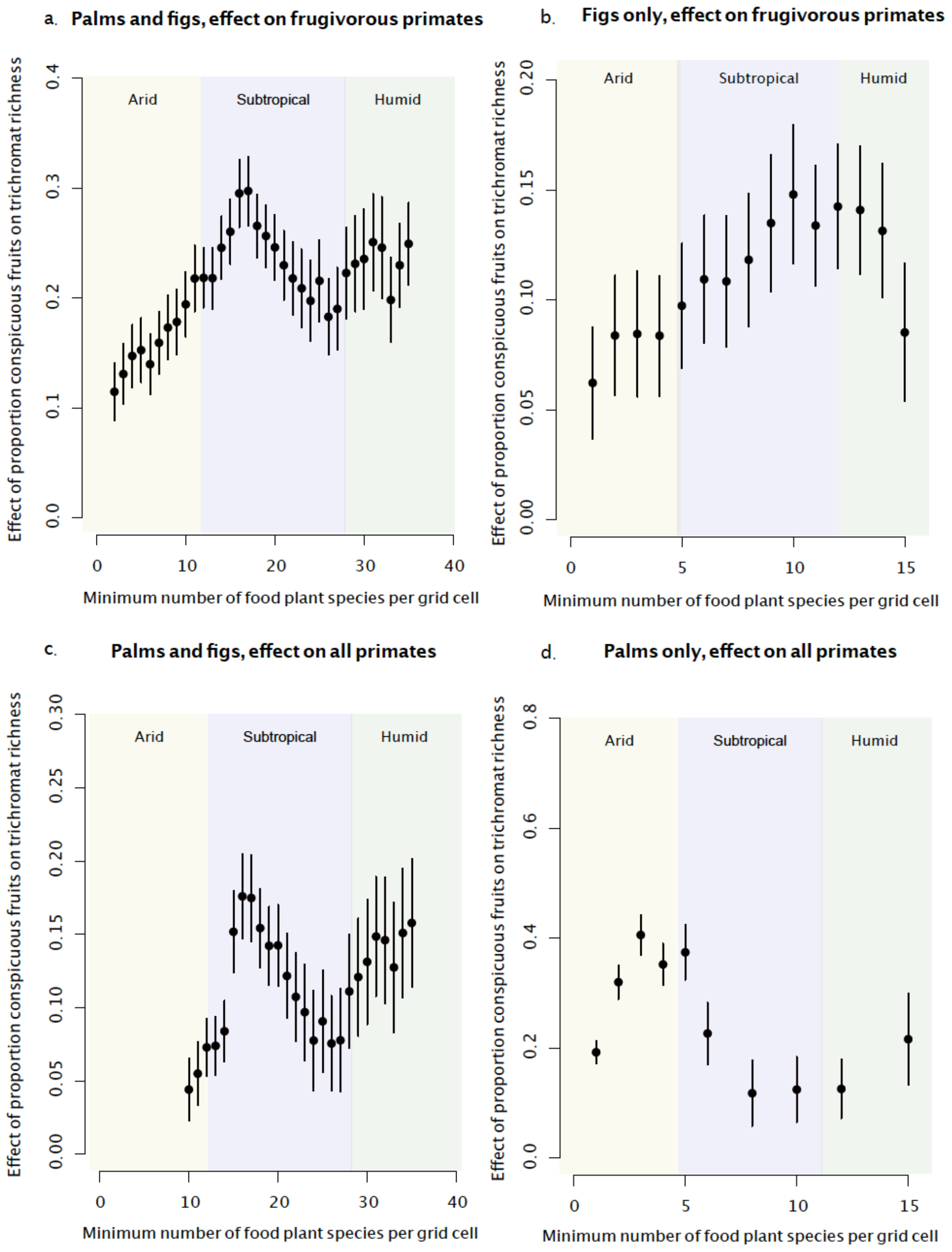

Figure S10: The progressive spatial delimitation of regions and climate zones based on minimum number of palm food plant species per grid cell. The African structural equation model (SEM) analyses were repeated on datasets including grid cells with a minimum of 2 to a minimum of 17 palm food plant species. This illustrates the progressive spatial delimitation of subtropical regions, eventually only including the most species-rich places in the humid tropics. Colours illustrate the biogeographical regions defined by Linder et al. (2012) which also largely correspond to main climatic zones following the Köppen-Geiger climate classification. The black dots indicate the centroids of grid cells included in the respective analysis.

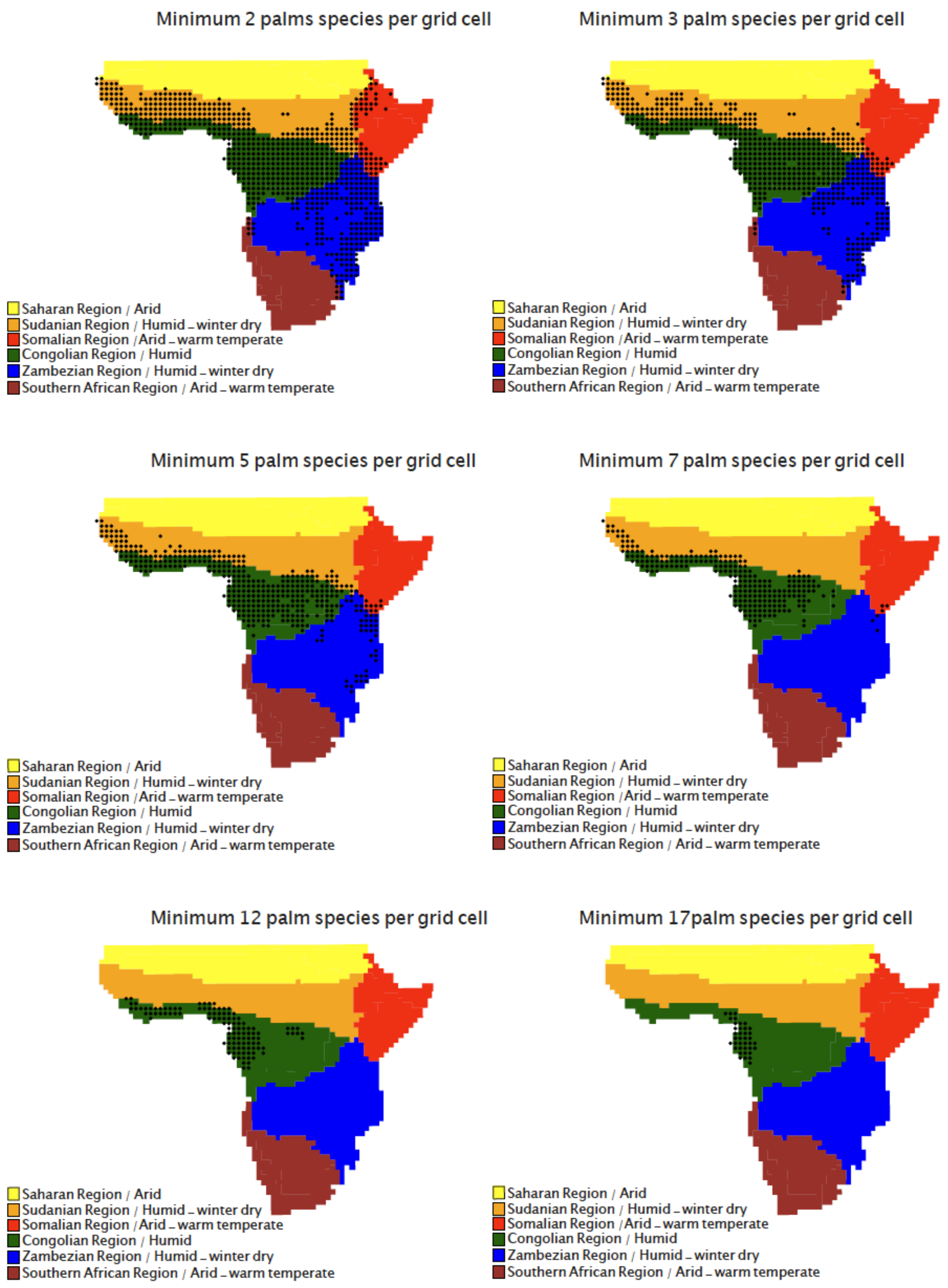

Figure S11: The global evolution of polymorphic and trichromatic colour vision in primates and conspicuous fruit colours in palms. Ancestral state reconstructions by stochastic character mapping of polymorphic/trichromatic colour vision and conspicuous fruits in primates and palms, respectively. The red/yellow/grey colour reflects the probability of the character states at the ancestral nodes and branches of the phylogenies. Polymorphic colour vision (a, in yellow) evolved at least two times independently: once ca. 30–40 million years ago (Ma) in the American monkeys and then possibly several times at ca. 20 Ma in the prosimians. Polymorphic vision preceded the evolution of routine trichromatic colour vision (a, in red). Routine trichromatic colour vision evolved twice independently within primates: once ca. 20–30 million years ago (Ma) in the African and Asian monkeys (Cercopithecidae), gibbons (Hylobatidae) and the great apes (Hominidae), and once ca. 15 Ma in the American howler monkeys (*Alouatta*). Conspicuous fruits (b, right) of palms evolved many times independently, and are at least ca. 40 million years old, but may be as old as the ancestral palm (ca. 116 Ma). Conspicuous fruits were certainly present ca. 40 Ma in the ancestors of African *Laccosperma, Eremospatha*, *Oncocalamus* and, more recently, in several lineages of *Raphia.* The evolution of conspicuous fruits therefore coincided with the evolution of polymorphic colour vision but preceded the evolution of routine trichromacy. Pictures illustrate primates with routine trichromatic colour vision (c) and conspicuous palm fruits (d–g): (c) *Pan troglodytes* feeding on the palm *Phoenix reclinata* (Photo credit: M. McLennan / Bulindi Chimpanzee & Community Project), (d) *Areca insignis* (photo credit: J. Dransfield), (e) *Hydriastele beguinii* (photo credit: W. Baker), (f) *Drymophloeus litigiosus* (photo credit: J. Dransfield), (g) *Dypsis spicata* (photo credit: J. Dransfield). Example genera of trichromatic primates and conspicuous palm fruits are indicated at the tips of the phylogenetic trees. The primate phylogeny was obtained from (Faurby and Svenning, 2015) and the palm phylogeny from (Onstein et al., 2017) (based on Faurby et al., 2016)

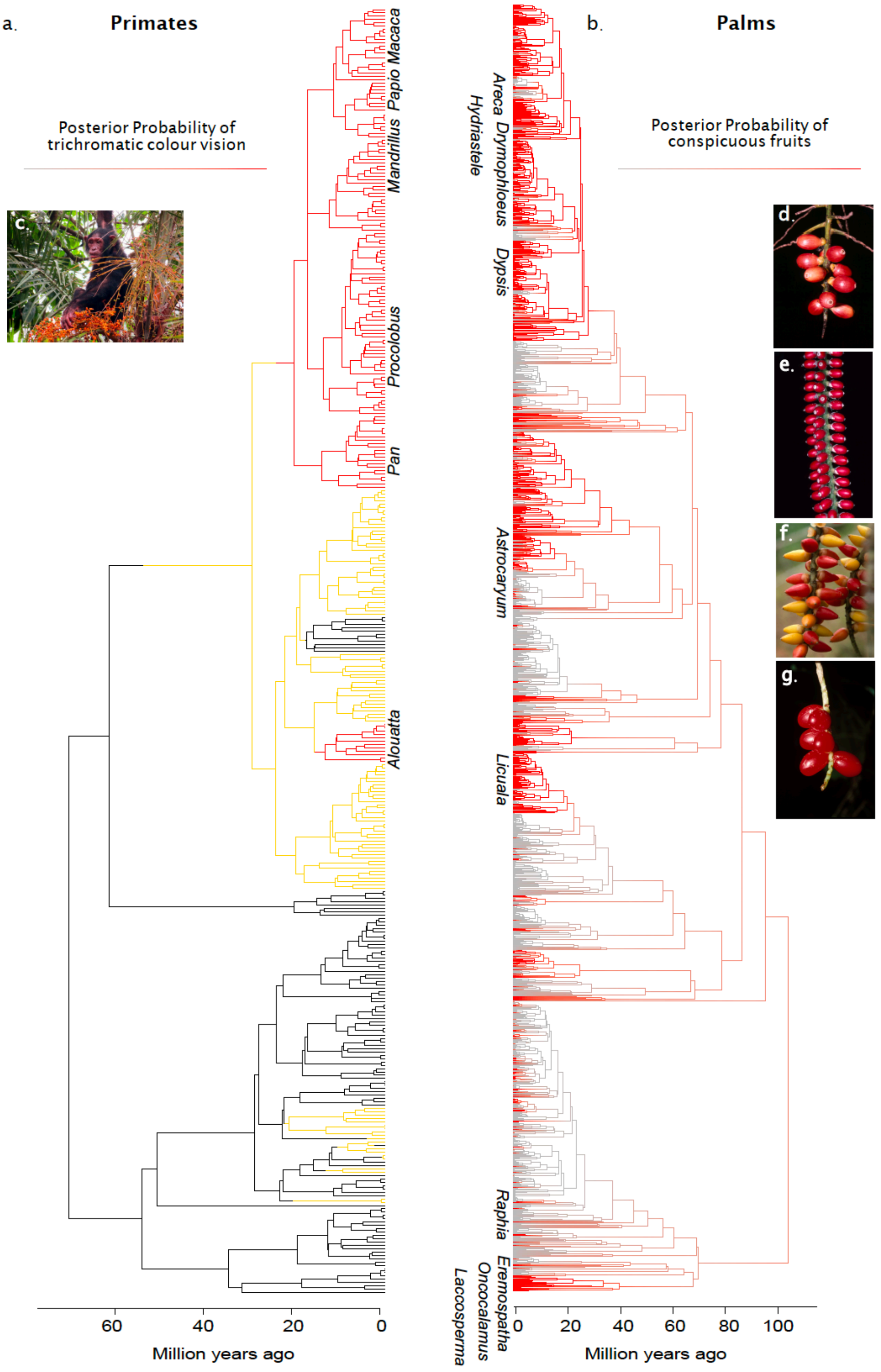

a.
Primates
Posterior Probability of
trichromatic colour vision
c.
Macaca
Papio
Mandrillus
Procolobus
Pan
Alouatta
60
40
20
0
Million years ago
b.
Palms
Posterior Probability of
conspicuous fruits
Areca
Hydriastele
Dymophloeus
Dypsis
Astrocaryum
Licuala
Raphia
Eremospatha
Oncocalamus
Laccosperma
0
20
40
60
80
100
Million years ago
d.
e.
f.
g.

Figure S12: Diversity changes of trichromatic and polymorphic primates through geological time in Africa, including Madagascar. The pie charts reflect ancestral nodes in the phylogeny of African primates and their probability to have trichromatic or polymorphic vision (red or yellow colours in pie-charts, obtained from ancestral state reconstructions). Non-trichromatic primates (dichromats) show a rapid radiation on Madagascar, which makes this figure different from that focused on mainland Africa only (see Fig. 4e).

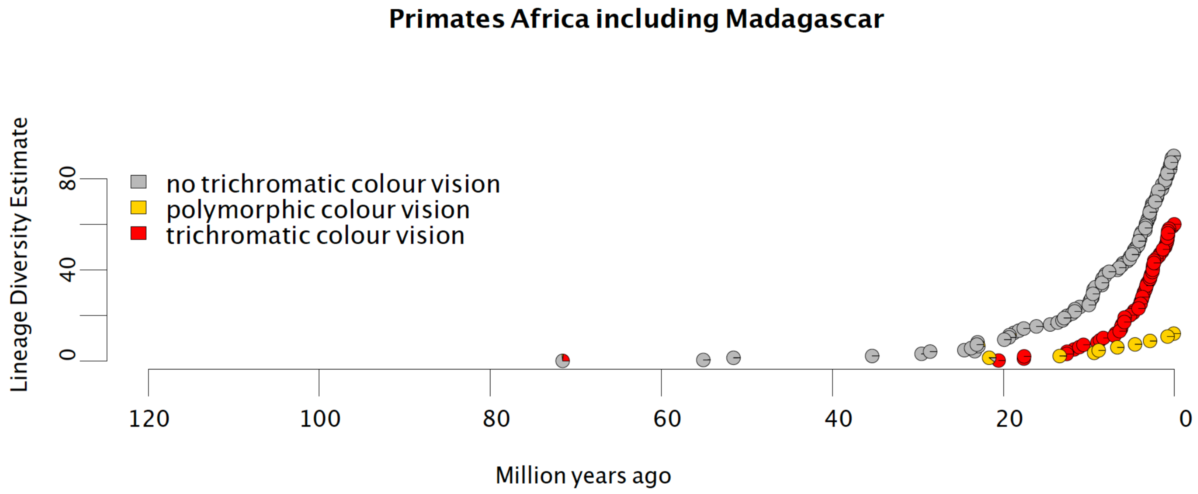

Figure S13: Diversity changes of conspicuous fig fruits through geological time in mainland Africa. The pie charts reflect ancestral nodes in the phylogeny of African figs and their probability to have conspicuous fruits (red colour in pie-charts, obtained from ancestral state reconstructions). Similar to palms (Fig. 4f), figs with conspicuous fruits show rapid diversity increases at ca. 10 million years ago as compared to figs with non-conspicuous fruits (grey colour in pie-charts). This happens in parallel with the radiation of trichromats in Africa (Fig. 4e).

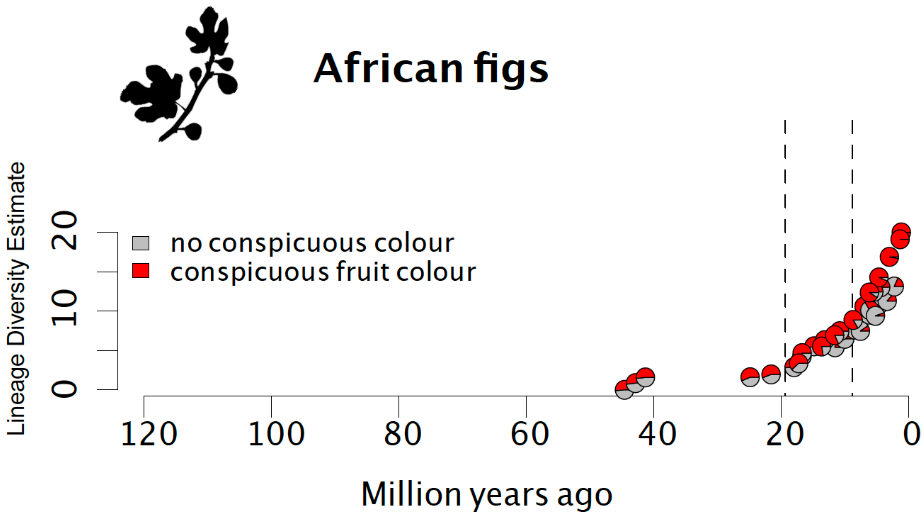

Figure S14: Diversification rates for polymorphic and trichromatic primates and palms with conspicuous fruits. We used Multiple State Speciation and Extinction (MuSSE) and Binary State Speciation and Extinction (BiSSE) models to model speciation, extinction, and transition rates of trichromatic/polymorphic colour vision vs. non-trichromatic/polymorphic colour vision (a) and of conspicuous fruits vs. non-conspicuous fruits (b). The MuSSE and BiSSE models jointly estimate speciation, extinction and transition rates of a trait with multiple trait states by using dated phylogenetic trees, and trait states assigned to the species at the tips of the trees. We fitted eight MuSSE or BiSSE models with decreasing complexity (parameters) and selected the best-fitting models based on likelihood-ratio tests under a Chi-square distribution and the Akaike Information Criterion (AIC). These models included constraints on speciation, extinction and transition rates between trait states. Maximum likelihood was used to optimize full and constrained models, and a Bayesian MCMC was run for the best-fitting models (constrained extinction and transition rates for primates and constrained speciation rates for palms) for 1000 generations on the maximum clade credibility trees. We show the posterior distribution of the diversification rates (speciation – extinction rates), indicating that trichromatic primates have the fastest diversification rates followed by polymorphic primates and non-trichromatic/non-polymorphic primates (a). Palm lineages with conspicuous fruits have faster diversification rates than palms with non-conspicuous fruits (b). Significance is indicated by the fact that the 95% Bayesian credibility intervals between parameter states do not overlap. The analyses were performed using the 'make.bisse' and 'make.musse' functions in the 'diversitree' R package (FitzJohn, 2012).

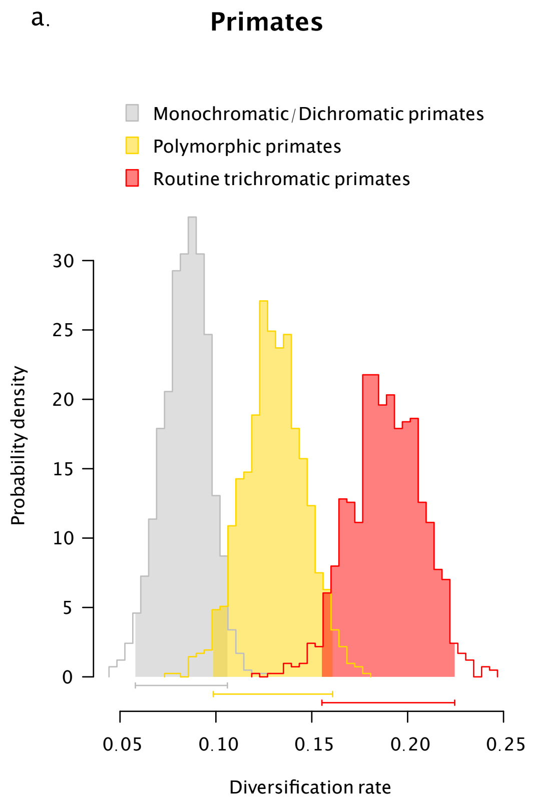

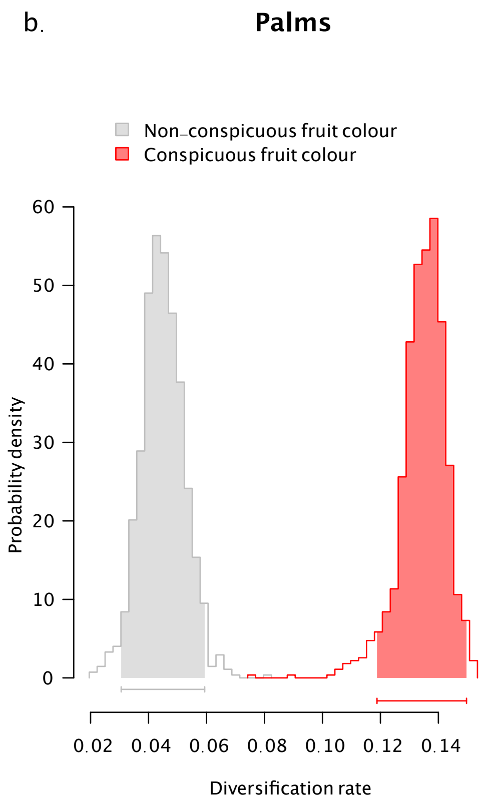

Figure S15: Diversity changes of folivorous and frugivorous primates in mainland Africa through geological time. The pie charts reflect ancestral nodes in the phylogenies and their probability to have the focal trait (red colour, obtained from ancestral state reconstructions): (a) folivory or (b) frugivory. In mainland Africa (i.e., excluding Madagascar), rapid diversity increases of frugivores happened ca. 10 million years ago (Ma) onward, compared to more gradual diversification of non-frugivorous African primates. In contrast, both folivorous and non-folivorous primates diversified rapidly from ca. 10 Ma onward, and this increase is thus unlikely to be related to this trait (i.e., folivory).

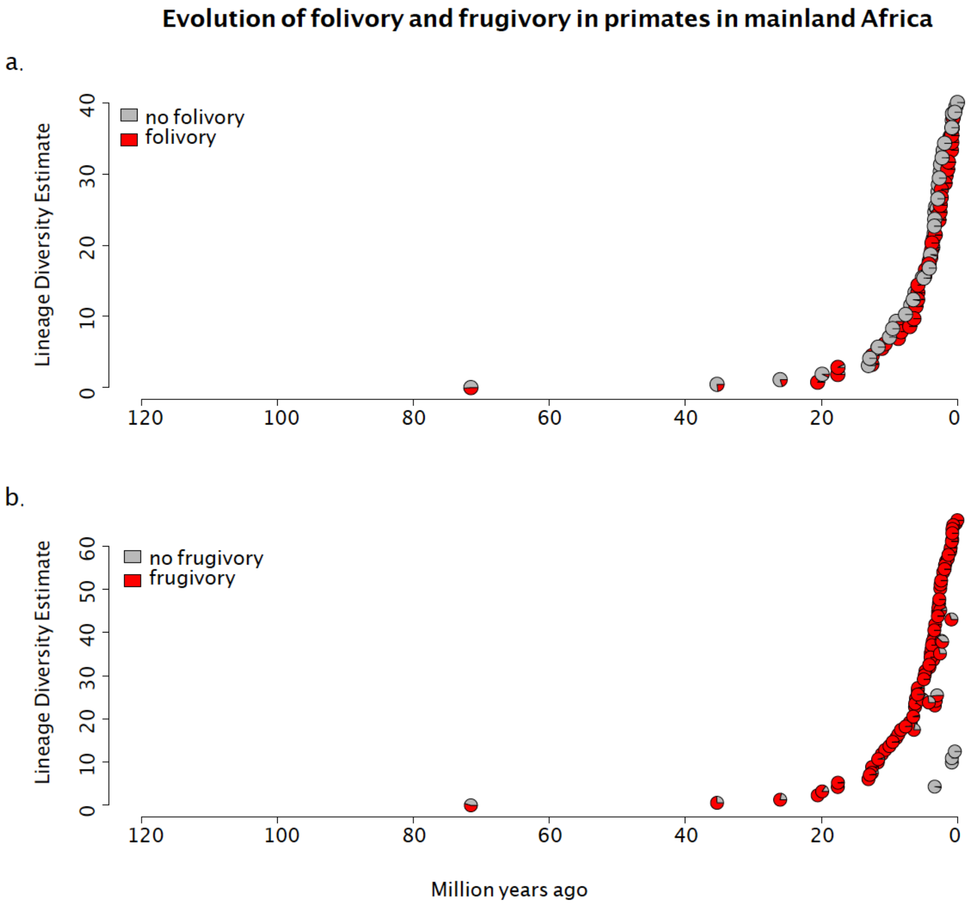

Figure S16: Fruit colour experiment. Visualization of measurements of red (R), green (G) and blue (B) and corresponding colours in the reflectance spectra of palm fruits (n = 18 species). Conspicuous and non-conspicuous refers to the classification of fruits following literature descriptions.

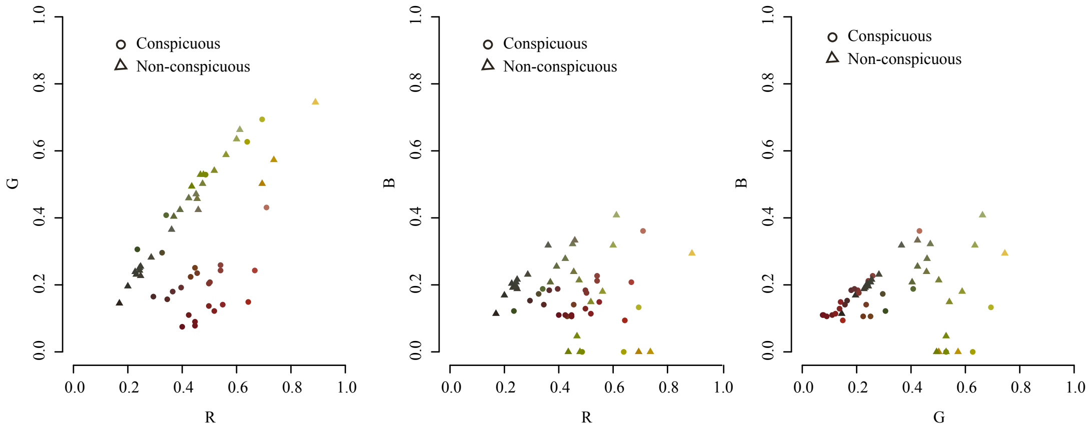

Figure S17: Global species richness of palms with conspicuous or non-conspicuous fruits. Maps depict the global distribution and variation of species richness of palm species with (a) conspicuous fruit colours and (b) non-conspicuous fruit colours. Only palm species with fruit colours known to be eaten by primates are included (brown, green, orange, yellow, red and purple fruits). Values are quantified at the spatial resolution of botanical countries. Grey colours indicate countries where no primates and / or < 2 palm species occur.

a.

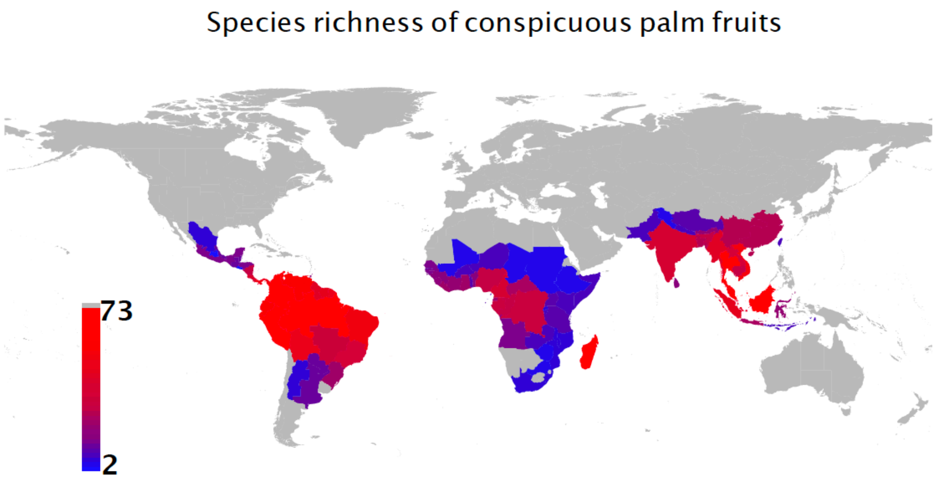

b.

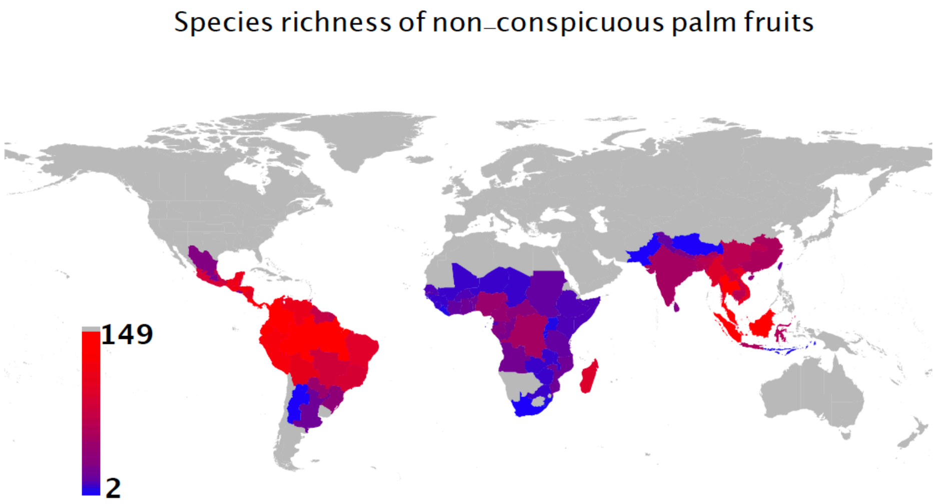

Supplementary references

Bivand, R. S. & Wong, D. W. S. (2018) Comparing implementations of global and local indicators of spatial association. *TEST,* **27,** 716–748.

Dominy, N. J., Svenning, J.-C. & Li, W.-H. (2003) Historical contingency in the evolution of primate color vision. *Journal of Human Evolution,* **44,** 25-45.

Faurby, S., Eiserhardt, W. L., Baker, W. J. & Svenning, J.-C. (2016) An all-evidence species-level supertree for the palms (Arecaceae). *Molecular Phylogenetics and Evolution,* **100,** 57-69.

Faurby, S. & Svenning, J.-C. (2015) A species-level phylogeny of all extant and late Quaternary extinct mammals using a novel heuristic-hierarchical Bayesian approach. *Molecular Phylogenetics and Evolution,* **84,** 14-26.

FitzJohn, R. G. (2012) Diversitree: comparative phylogenetic analyses of diversification in R. *Methods in Ecology and Evolution,* **3,** 1084-1092.

Fleming, T. H. & Kress, W. J. (2013) *The ornaments of life: coevolution and conservation in the tropics.* University of Chicago Press.

Gainsbury, A. M., Tallowin, O. J. S. & Meiri, S. (2018) An updated global data set for diet preferences in terrestrial mammals: testing the validity of extrapolation. *Mammal Review,* **48,** 160-167.

Galán-Acedo, C., Arroyo-Rodríguez, V., Andresen, E. & Arasa-Gisbert, R. (2019) Ecological traits of the world's primates. *Scientific Data,* **6,** 55.

Govaerts, R. & Dransfield, J. (2005) *World checklist of palms.* Royal Botanic Gardens, Kew, UK.

Grace, J. B., Schoolmaster, D. R., Guntenspergen, G. R., Little, A. M., Mitchell, B. R., Miller, K. M. & Schweiger, E. W. (2012) Guidelines for a graph-theoretic implementation of structural equation modeling. *Ecosphere,* **3,** art73.

Huelsenbeck, J. P., Nielsen, R. & Bollback, J. P. (2003) Stochastic mapping of morphological characters. *Syst Biol,* **52,** 131-158.

Kissling, W. D., Balslev, H., Baker, W. J., Dransfield, J., Göldel, B., Lim, J. Y., Onstein, R. E. & Svenning, J.-C. (2019) PalmTraits 1.0, a species-level functional trait database of palms worldwide. *Scientific Data,* **6,** 178.

Kissling, W. D. & Carl, G. (2008) Spatial autocorrelation and the selection of simultaneous autoregressive models. *Global Ecology and Biogeography,* **17,** 59-71.

Kissling, W. D., Dalby, L., Fløjgaard, C., Lenoir, J., Sandel, B., Sandom, C., Trøjelsgaard, K. & Svenning, J.-C. (2014) Establishing macroecological trait datasets: digitalization, extrapolation, and validation of diet preferences in terrestrial mammals worldwide. *Ecology and Evolution,* **4,** 2913-2930.

Kissling, W. D., Field, R. & Böhning-Gaese, K. (2008) Spatial patterns of woody plant and bird diversity: functional relationships or environmental effects? *Global Ecology and Biogeography,* **17,** 327-339.

Kissling, W. D., Rahbek, C. & Böhning-Gaese, K. (2007) Food plant diversity as broad-scale determinant of avian frugivore richness. *Proceedings of the Royal Society of London B: Biological Sciences,* **274,** 799-808.

Legendre, P. & Legendre, L. (1998) *Numerical ecology.* Elsevier, Amsterdam, The Netherlands.

Linder, H. P., de Klerk, H. M., Born, J., Burgess, N. D., Fjeldså, J. & Rahbek, C. (2012) The partitioning of Africa: statistically defined biogeographical regions in sub-Saharan Africa. *Journal of Biogeography,* **39,** 1189-1205.

Mittermeier, R. A., Rylands, A. B., Wilson, D. E. & Martinez-Vilalta, A. (2013) *Handbook of the mammals of the world: Primates.* Lynx Edicions, Barcelona.

Onstein, R. E., Baker, W. J., Couvreur, T. L. P., Faurby, S., Svenning, J.-C. & Kissling, W. D. (2017) Frugivory-related traits promote speciation of tropical palms. *Nature Ecology & Evolution,* **1,** 1903-1911.

Regan, B. C., Julliot, C., Simmen, B., Viénot, F., Charles–Dominique, P. & Mollon, J. D. (2001) Fruits, foliage and the evolution of primate colour vision. *Philosophical Transactions of the Royal Society of London. Series B: Biological Sciences,* **356,** 229-283.

Shanahan, M., So, S., Gompton, S. G. & Corlett, R. (2001) Fig-eating by vertebrate frugivores: a global review. *Biological Reviews,* **76,** 529-572.

Sinnott-Armstrong, M. A., Downie, A. E., Federman, S., Valido, A., Jordano, P. & Donoghue, M. J. (2018) Global geographic patterns in the colours and sizes of animal-dispersed fruits. *Global Ecology and Biogeography,* **27,** 1339-1351.

Wilman, H., Belmaker, J., Simpson, J., de la Rosa, C., Rivadeneira, M. M. & Jetz, W. (2014) EltonTraits 1.0: Species-level foraging attributes of the world's birds and mammals. *Ecology,* **95,** 2027-2027.

Zhang, Q., Onstein, R. E., Little, S. A. & Sauquet, H. (2018) Estimating divergence times and ancestral breeding systems in *Ficus* and Moraceae. *Annals of Botany,* **123,** 191–204.
